# *Pleurotus sajor-caju*-Mediated Synthesis of Silver and Gold Nanoparticles Active against Colon Cancer Cell Lines: A New Era of Herbonanoceutics

**DOI:** 10.3390/molecules25133091

**Published:** 2020-07-07

**Authors:** Vivek K. Chaturvedi, Navneet Yadav, Neeraj K. Rai, Noura H. Abd Ellah, Raghvendra A. Bohara, Ibrahim F. Rehan, Najat Marraiki, Gaber El-Saber Batiha, Helal F. Hetta, M. P. Singh

**Affiliations:** 1 Centre of Biotechnology, University of Allahabad, Prayagraj 211002, India; vivekchaturvedi2013@gmail.com; 2 Department of Physics, University of Allahabad, Prayagraj 211002, India; navaneetyadav840@gmail.com; 3 Department of Biotechnology, Central University of South Bihar, Gaya 824236, India; neerajrai2601@gmail.com; 4 Department of Pharmaceutics, Faculty of Pharmacy, Assiut University, Assiut 71526, Egypt; nora.1512@yahoo.com or; 5 Centre for Interdisciplinary Research D.Y. Patil University, Kolhapur 416006, India; raghvendrabohara@gmail.com; 6 Department of Husbandry and Development of Animal Wealth, Faculty of Veterinary Medicine, Menofia University, Shebin Alkom, Menofia 32511, Egypt; ibrahim.rehan@vet.menofia.edu.eg; 7 Department of Botany and Microbiology, College of Science, King Saud University, Riyadh 11451, Saudi Arabia; Najat@ksu.edu.sa; 8 Department of Pharmacology and Therapeutics, Faculty of Veterinary Medicines, Damanhour University, Damanhour 22511, Egypt; gaberbatiha@gmail.com; 9 Department of Medical Microbiology and Immunology, Faculty of Medicine, Assiut University, Assiut 71515, Egypt; 10 Department of Internal Medicine, University of Cincinnati College of Medicine, Cincinnati, OH 45267, USA

**Keywords:** herbonanocuetics, *Pleurotus sajor-caju*, nanoparticles, HCT-116, cytotoxic, ROS, transmission electron microscopy, anticancer activity

## Abstract

Herbal medicines are widely used worldwide and much appreciated because of their fewer side effects and the ability to fight diseases at the root cause. Active ‘phyto’ ingredients require a scientific approach and a mechanism to distribute components at the target site for better therapeutic results. Nanotechnology, on the other hand, has created new hope for cancer treatment but is still far from being proven in clinical settings. This article combines a unique approach to synthesis with the use of *Pleurotus sajor-caju*, followed by microwave irritation of silver and gold nanoparticles that ensures the capping of the active phyto ingredient and further enhances the effects of nanomedicine to fight colon cancer, thus opening a new era of what we call herbonanoceutics. The article also compares the characteristics and properties of silver (Au) and gold (Ag) nanoparticles synthesized by an in house developed novel microwave-assisted rapid green synthesis method. The as-prepared Ag NPs and Au NPs were compared using ultraviolet-visible spectroscopy (UV-Vis), Fourier transform infrared (FTIR) spectroscopy, X-ray diffraction (XRD), transmission electron microscopy (TEM), and energy dispersive spectroscopy (EDS). Our comparative study revealed that both assemblies display face-centred cubic structures (FCCs) and are nanocrystalline in nature. The advantage of the approach was that the sizes of gold and silver were identical in range with a similar distribution pattern. This has helped us to study the activity against colon cancer cell line (HCT-116) without incoherence since size plays a key role in the application. More specifically, morphological changes, cell viability, the production of reactive oxygen species (ROS) and the fragmentation of DNA have been further reported to assess better the results obtained with the two metals. Our results suggest that the newly adopted synthesis method may ensure the dual benefits from phyto ingredients which further enhances the effectiveness of advanced nanomedicine.

## 1. Introduction

In the past decade, nanoparticle (NP)-based approaches have emerged for the diagnosis and treatment of cancer [1,2]. Colon cancer is one of the most common gastrointestinal malignancy-related causes of death worldwide, with very few or no treatment options [3]. Recently herbal medicine has shown a new hope to fight against colon cancer. However, due to non-specificity and short half-life, much success has not been achieved. Nanoparticles (NPs)-based therapy can be used in the treatment of colon cancer as an alternative to conventional therapy (surgery, radiation and chemotherapy), which are expensive and less effective, with a high degree of side effects. Due to enhanced permeability and retention effect (EPR), and thanks to the tumour microarchitecture, nanotechnology certainly has created a new hope in the treatment regimen to fight against cancer.

Even though a large number of publications have proved the effectiveness of nanomedicine, it is still far away from clinical translation. One possible reason is that too much dependence upon the intrinsic properties and makes it far more complicated, which in terms of clinical setting is impossible to replicate. To overcome this limitation and to make nanomedicine more applicable at the clinical level, the use of herbonanoceutics methods of synthesis may be beneficial. Herbonanoceutics is the herbal-mediated synthesis of nanoparticles which ensures the synthesis of nanoparticles and at the same time ensures the capping of herbal drugs modifying the surface of nanoparticles which enhances the activity of the nanomedicine. In short, this system ensures targeting at the desired site and ensures the delivery of the active phytoingredient to enhance the overall effect of the therapy. From the literature it can be seen that many green-mediated syntheses have been reported, but capping of active ingredients on the surface of nanoparticles and uniformity in size and shape is not frequently discussed.

Recently various types of metal NPs have been synthesized biologically by reducing and capping of metal ions using extracts of macrofungi such as *Pleurotus ostreatus, Lentinus edodes, Ganoderma lucidum, and Grifola frondosa* [4,5]. Vigneshwaran et al. [6] and Gade et al. [7] have synthesized silver-protein NPs and silver NPs, respectively, using *P. sajor-caju* extracts. Vigneshwaran used the mushroom waste for synthesis. In another study he also discussed synthesis of silver NPs by various bioagents including *P. sajor-caju* and also compared their biomedical applications [8,9]. Musa et al. [10] synthesized the silver NPs by *Pleurotus sajor-caju* and showed its antifungal effects against *Candida albicans*.

However, all these syntheses involve either use of plant extract or microbial cells with no further treatment, which makes them far away from practical applicability. Recently, we have developed an in-house synthesis method that uses biological extracts, accompanied by a highly structured microwave irradiation process to successfully reduce and cap Au and Ag nanoparticles with the active ingredients of *Pleurotus sajor-caju* extracts. Our extracts of biological origin contain several kinds of primary and secondary metabolites that can be ketones, terpenes, flavones, aldehydes and amides, which play vital roles in the reduction of nanoparticles and the resulting therapeutic properties.

We also describe a comparative study between the activity of gold and silver NPs, in particular in colon cancer cells. Currently, gold and silver nanoparticles are used widely and it is difficult to choose amongst them in biomedical applications. As far as biomedical applications are concerned, a comparison between different nanosystems should be made at the same stage in order to obtain proper comparisons. Most of the green synthesis methods developed apply to the synthesis of one specific metal. They do not yield similar results when used for other metal synthesis, other than those mentioned. Therefore, this study aimed to synthesize a novel in house developed Ag NPs, and Au NPs by using *Pleurotus sajor-caju* extract (Ag NPs and Au NPs), followed by microwave assisted irridiation. Our study also explores a comparative study of the cytotoxic effect of these NPs on a human colorectal carcinoma (HCT-116) cell line. Furthermore, the cytotoxicity caused by mushroom extract (ME), Ag NPs, and Au NPs was addressed with different assays such as morphology assay, MTT assay, and ROS generation providing a path for the next generation of herbonanoceutics.

## 2. Results

### 2.1. Characterization of Ag NPs and Au NPs

#### 2.1.1. UV-Visible Spectroscopy

**Figure 1:**
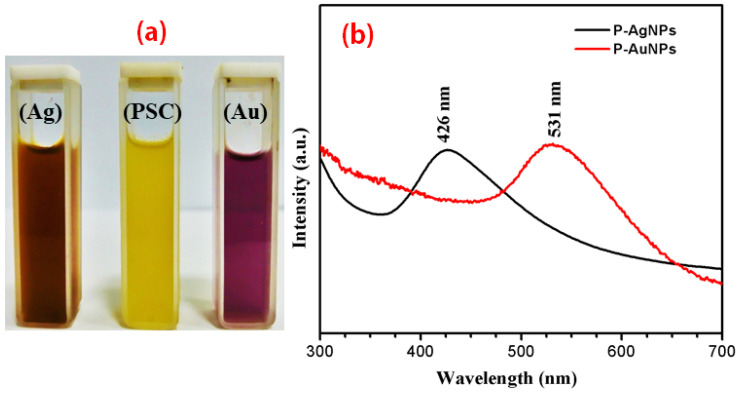
The UV-visible spectra of synthesized nanoparticles. (**a**) Yellowish aqueous PS extract, brown color Ag NPs and pinkish violet Au NPs, respectively, (**b**) UV-visible spectra of Ag NPs and Au NPs.

After the addition of precursors, extract and followed by optimized microwave irritation, we observed that there was a shift in color from the initial colorless solutions to dark brown and pinkish-purple transparent colloidal solution, indicating the synthesis of Ag NPs and Au NPs, respectively, as seen in Figure 1a. We also tried to synthesize the NPs without using microwave irritation (data not shown), but we observed faint color changes which were very slow (after 12 h). The the visible UV-absorption spectra of the biosynthesized metal colloids showed a single absorption band in which the maximum wavelength was associated with the surface plasmon resonance (SPR) of the metal NP observed at 426 and 531 nm for Ag NPs and Au NPs, respectively, as shown in Figure 1b.

#### 2.1.2. X-ray Diffraction (XRD) Analysis

**Figure 2:**
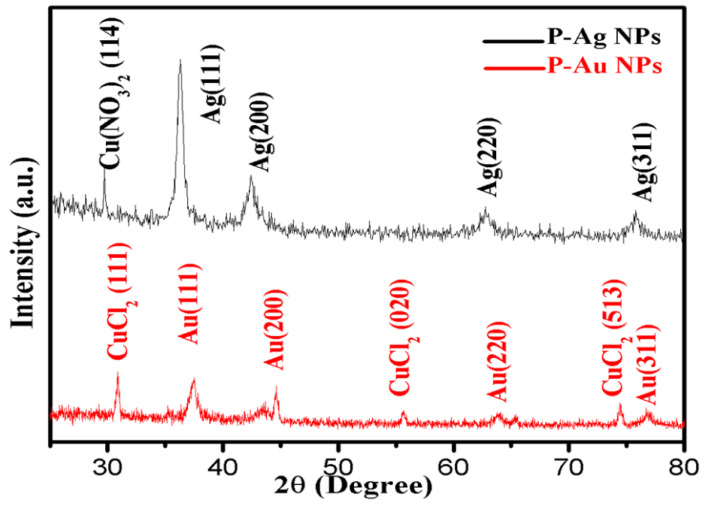
XRD spectra of biosynthesized Ag NPs and Au NPs confirms the pure phase formation of Au and Ag nanoparticles.

The XRD peaks of biosynthesized Ag NPs at 2θ were observed at 37.32°, 43.31°, 63.73° and 76.68° and assigned to the lattice plane of Ag(111), Ag(200), Ag(220) and Ag(311), respectively, which confirms the face-cantered cubic (FCC) structure, which is similar to the standard 2θ of Ag metal (JCPDS No. 04-0783). The average crystallite size is 16.82 nm. A small peak at 2θ = 30.64° was also observed which may be due to the presence of another metal, i.e., Cu, Cd, Zn or Ni, in the ME. The XRD peaks of the Au NPs obtained at 2θ were at 37.53°, 44.59°, 64.05° and 76.78°, and for these values of 2θ mentioned in Table 1, the lattice plane indexes of Au are Au(111), Au (200), Au (220) and Au (311). All these findings are similar to the standard of Au metal (JCPDS No. 04-0784). Moreover, some new peaks of CuCl_2_ were also observed in the XRD pattern of Au NPs but not in Ag NPs, which might be due to the interaction of chloride ions present in Au salt with Cu metal, present in mushrooms as reported in Mallikarjuna et al. [11]. The values of peaks were 2θ = 30.89°, 55.71°, 74.43° and plane of face CuCl_2_ (111), CuCl_2_ (020) and CuCl_2_ (513), respectively (JCPDS No. 79-1635). The average crystalline size of Au NPs measured 16.90 nm (Figure 2 and Table 1).

#### 2.1.3. Dynamic Light Scattering (DLS)

**Figure 3:**
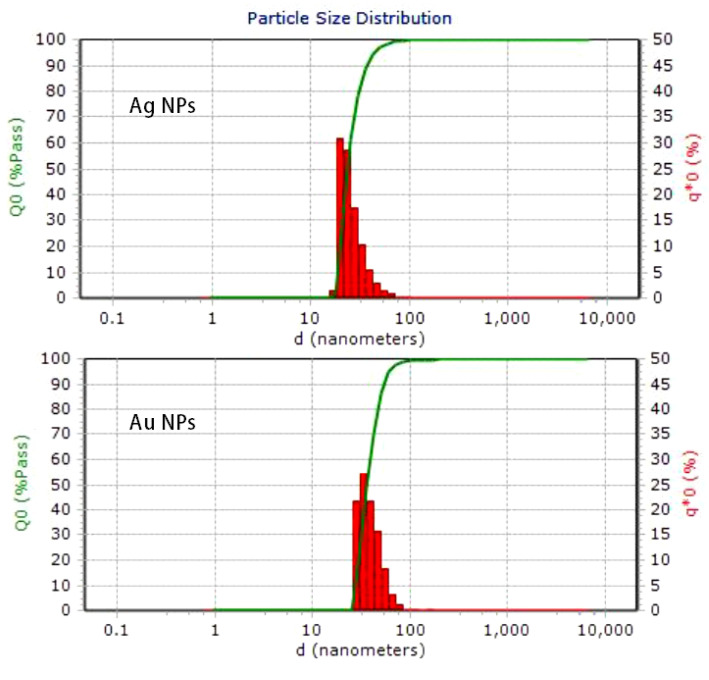
Particle size distribution of silver and gold nanoparticles.

As seen in Figure 3, the DLS method was used to determine the hydrodynamic diameter of the biosynthesized nanoparticles. The obtained sizes were 23 nm (silver) and 37 nm (gold). In the DLS measurements the size of the nanoparticle appeared larger than in the TEM micrographs because of the solvent layers present around the nanoparticles.

#### 2.1.4. Fourier Transforms Infrared Spectroscopy (FTIR)

**Figure 4:**
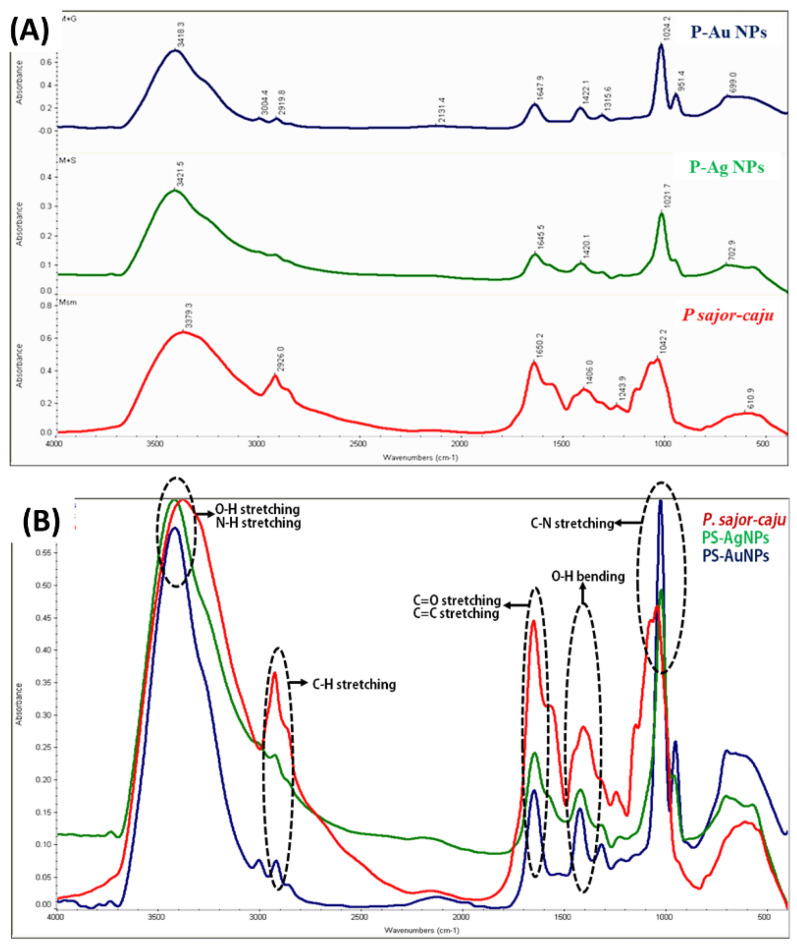
(**A**) FTIR absorption spectra of PS extract, Ag NPs and Au NPs., which confirm the capping of the phyto extract on the surface of the synthesized nanoparticles (**B**) FTIR overlay with stacking peaks of PS extract, Ag NPs and Au NPs with their respective functional groups.

FTIR was used to confirm the capping of the phyto extract on the surface of the synthesized nanoparticles. Our FTIR results indicated the presence of active functional group from the PS extract on the surface of the nanoparticles. The region from 1000–500 cm^−1^ is known as fingerprint region in which the peaks are usually due to metal oxide bonds. In contrast, the peaks beyond the 1000 cm^−1^ range (i.e., 4000–1000 cm^−1^) likely arise due to the bending and stretching vibrations of water molecules 3379.3, 3421.5 and 3418.3 cm^−1^ respectively, these peaks are characteristic of O-H stretching and N-H stretching in amide groups. Moreover, several other characteristic vibrational peaks were observed, i.e., 1650.2, 1645.5 and 1674 cm^−1^, which can be attributed to capping of the active group on the surface during the synthesis of Ag NPs and Au NPs, which are characteristic of alkenes (C=C stretch) and amide stretching (C=O stretching) (Figure 4). 

We also observed the presence of aromatic compounds on the surface of nanoparticles (C=C stretching). The peaks 1406.0, 1420.1 and 1422.1 cm^−1^ and 1021.7 to 1042.2 cm^−1^ confirmed the presence of alcohol (O-H bending) and amine (C-N stretching) functions, respectively, as listed in Table 2. All these peaks confirm the capping of active ingredients like (polysaccharide β-glucans) on the surface of nanoparticles makes them enriched and effective.

#### 2.1.5. Scanning Electron Microscopy (SEM) and Transmission Electron microscopy (TEM) Analysis

**Figure 5:**
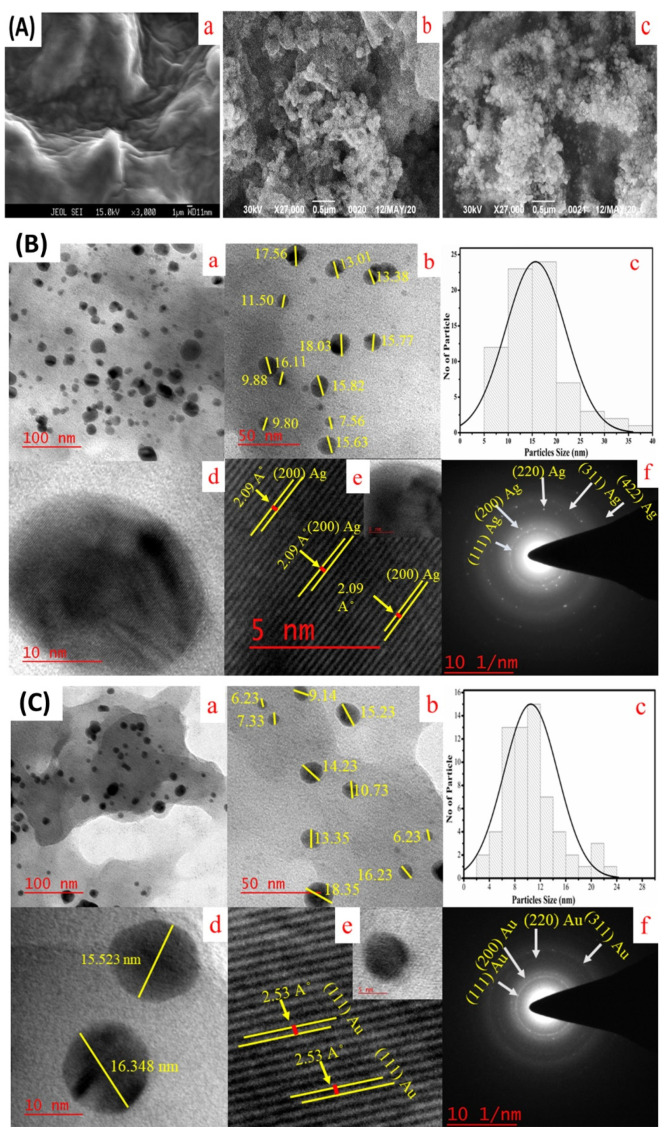
(**A**) SEM images of Ag NPs and Au NPs synthesized from aqueous extract of oyster mushroom *P. sajor-caju*. (**a**) Aqueous extract of oyster mushroom *P. sajor-caju*; (**b**) *P. sajor-caju* mediated synthesized Ag NPs and (**c**) Au NPs. (**B**) TEM images and size distribution histogram of biosynthesized Ag NPs (**a** to **f**) in which the scale bar represents at 100 nm (**a**), 50 nm (**b**), particle size histogram analysis of Ag NPs (**c**), 10 nm (**d**), and at 5 nm high-resolution TEM (HR-TEM) image of a single Ag NPs respectively with lattice fringes (**e**) and SAED pattern (**f**).; (**C**) TEM images and size distribution histogram of biosynthesized Au NPs (**a** to **f**) at 100 nm (**a**), at 50 nm (**b**), particle size histogram analysis of Au NPs (**c**), at 10 nm (**d**), at 5 nm HR-TEM image of an Au NPs respectively with lattice fringes (**e**) and SAED pattern (**f**).

The size and shape of the biosynthesized Ag NPs and Au NPs were further characterized by SEM and TEM. A SEM instrument was employed to analyse the surface structure of the nanoparticles that were formed. From Figure 5A it is evident that Ag NPs and Au NPs were spherical and *P. sajor-caju extract* was aggregated and capped over the silver and gold nanoparticles [12]. The TEM images illustrated that the Ag NPs are crystalline and spherical having an average diameter of 15–20 nm (Figure 5B). The histogram in Figure 5B(c) shows the homogeneous size distribution of Ag NPs is in the range of 10–16 nm. The calculated d-value from the lattice fringe pattern is 2.09 Å (Figure 5B(e)). The selected area electron diffraction (SAED) pattern of particles again depicted the polycrystalline nature of NPs from XRD data -with preferential orientation along (111) (200), (220), (311) lattice planes as reported by Shinde et al., [13] and the d-values of Ag NPs were 2.30, 1.96, 1.36 and 1.16 Å (Figure 5B(f)). The high-resolution TEM (HR-TEM) images of biosynthesized Au NPs show a spherical shaped structure having an average diameter of 16–18 nm (Figure 5C). The histogram of Au NPs showed the particle size distribution ranges from 4–22 nm (Figure 5C(c)). The calculated lattice fringe (*d*-values) was 2.35 Å (Figure 5C(e)). The SAED pattern of Au NPs confirms its polycrystalline nature and the *d*-values are 2.20, 1.98, 1.36, and 1.12 Å (Figure 5C(f)).

#### 2.1.6. EDS Analysis

**Figure 6:**
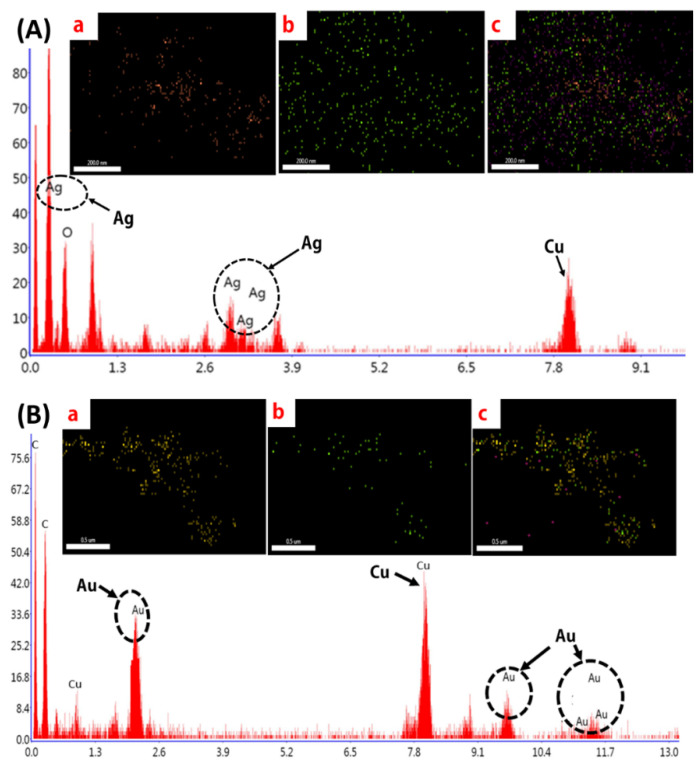
(**A**) EDX and elemental mapping of Ag NPs. (**a**). Uniform distribution silver NPs (**b**). oxygen, (**c**) Mixed elemental mapping of carbon, oxygen and silver NPs; (**B**) EDX and elemental mapping of Au NPs. (**a**). Gold NPs (**b**). oxygen, (**c**) Mixed elemental mapping of carbon, oxygen and gold NPs.

The EDS and elemental mapping of biosynthesized Ag NPs (Figure 6A) and Au NPs (Figure 6B) depict the peaks for Ag NPs and Au NPs with some other peaks which might be attributed to the capping of active ingredient on the surface of nanoparticles. The dispersal of Ag NPs and Au NPs in the nanofluid was also well and homogeneous. The EDS and specific elemental mapping of synthesized Au nanofluids revealed the presence of Au. Here again, additional peaks of copper and carbon were reported, which further confirms the presence of active ingredients on the surface of the nanoparticles.

### 2.2. Cytotoxicity Assessment of PS Extract, Ag NPs, and Au NPs against HCT-116 Cell Line

**Figure 7:**
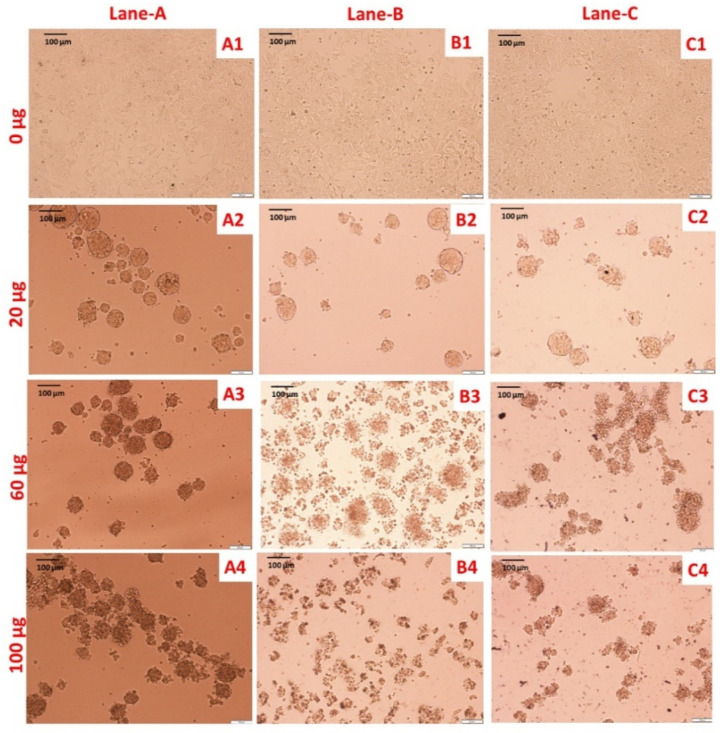
Morphological analysis of HCT-116 cell line at various concentration of PS extract, Ag NPs and Au NPs. Lane A, B and C shows exposure of aqueous extract of PS extract, Ag NPs and Au NPs. In lane A, untreated cells (**A1**), cells treated with different concentrations (20–100 μg/mL) of PS extract (**A2**–**A4**). In lane B, untreated cells (**B1**), cells treated with different concentrations (20–100 μg/mL) of Ag NPs (**B2**–**B4**). In lane C, untreated cells (**C1**), cells treated with different concentrations (20–100 μg/mL) of Au NPs (**C2**–**C4**) (scale bar  =  100 μm; magnification: 20×).

The HCT-116 colon cancer cell line was treated with PS extract and biologically synthesized Ag NPs and Au NPs at various concentrations for 24 h and cell viability was measured by a MTT colorimetric assay. We recorded morphological changes in treated cells in comparison with untreated cells (Figure 7).

**Figure 8:**
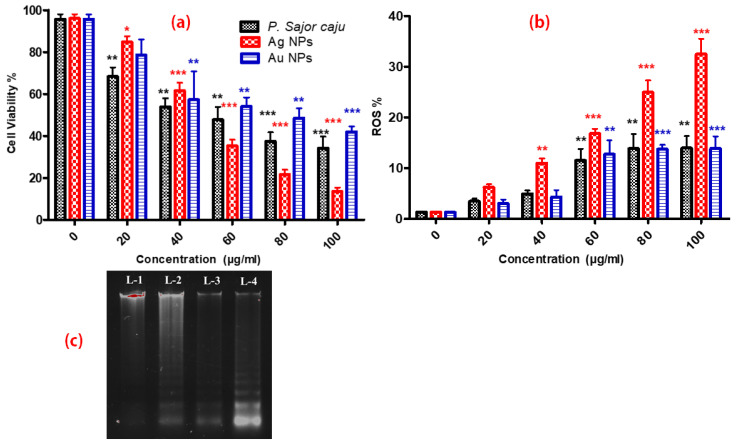
(**a**) Cell viability of HCT-116 cell line at various concentrations (20–100 μg/mL) of PS extract, Ag NPs and Au NPs. (**b**) Intracellular ROS production against HCT-116 cell line at various concentrations (20–100 μg/mL) of PS extract, Ag NPs and Au NPs. (**c**) DNA fragmentation assay using agarose gel electrophoresis. Lane 1- Control, Lane 2- aqueous PS extract, Lane 3- Au NPs, Lane 4- Ag NPs treated HCT-116 cell line. All data were analysed by one-way ANOVA using post hoc Dunnett’s test. *p* < 0.05 was considered to be statistically significant between exposed and control groups. The symbols *, *, *** show statistical significance using one-way ANOVA (*p* < 0.05, *p* < 0.01, *p* < 0.005, respectively), compared to positive control.

Viability of HCT-116 cells was inhibited by PS extract, Ag NPs and Au NPs with an IC_50_ value of 60, 50 and 80 μg/mL respectively (Figure 8a). It can be clearly seen from the IC_50_ values that extract successfully transferred to the surface of nanoparticles has anticancer potential and makes them more lethal in comparison. Overall, the cell viability was significantly decreased after 24 h of dosing as compared with control. Among all three samples tested, Ag NPs showed the highest cytotoxicity on HCT-116 cell lines. Moreover, between Au NPs and PS extract; PS extract showed better cytotoxicity. Our results clearly showed that the developed method was successful at transferring the active phyto constituents to the surface of nanoparticles. We also noted that the Ag NPs have more toxicity against the HCT-116 cell line, followed by PS extract and Au NPs. Our results support the observation made by Rahman et al. who stated that Ag NPs generate more ROS that can attack proteins causing oxidative stress resulting in an increment in permanent damage of protein integrity and functionality and also the presence of active constituent on the surface further enhanced the activity [14].

### 2.3. Assessment of ROS

The DCFH-DA staining procedure is an indirect method for assessing ROS generation by measuring intracellular free radicals. The present study showed that the addition of PS extract and biosynthesized Ag NPs and Au NPs caused cell death. The intracellular ROS production was significantly increased in treated cells as compared to the non-treated cells after 24 h. Figure 8b, clearly shows that the generation of ROS is more with Ag NPs as compared with PS extract and Au NPs. According Liou et al., when ROS level increase more than the threshold level cells undergoes to apoptosis. So, ROS generated by 20 and 40 μg/mL were below the threshold level and ROS generated by 60, 80 and 100 μg/mL were higher than threshold level that is why we can see the difference in the pattern of viability and ROS level [15].

### 2.4. DNA Fragmentation Assay

The assay was performed to investigate the effect of PS extract, Ag NPs and Au NPs on the human colon cancer cell line (HCT-116). To determine the effect of PS extract and synthesized NPs on DNA damage, HCT-116 cell line was treated with 100 µL of the effective concentration (based on MTT cell viability assay) of PS extract, Au NPs and Ag NPs sample for 24 h along with control i.e., lane 2, lane 3, lane four and lane 1, respectively. The results depicted that Ag NPs induced more cell death by cleaving the nuclear DNA of HCT-116 by forming a ladder pattern as compared with PS extract and Au NPs (Figure 8c). A similar type of result was justified by Gurunathan and co-workers, where cancer cell lines, which were treated with Ag NPs exhibit the formation of the DNA ladder [11].

## 3. Discussion

There is a common saying “medicines and foods have a common origin.” Mushrooms are the manifestation of this idea in constituting both a nutritionally functional food and a source of physiologically beneficial components with possible medicinal applications. It was found that the active component behind the anti-cancer activity of *Pleurotus* mushrooms species is its polysaccharide fraction, pleuran, which is an essential constituent of *Pleurotus* spp. This component has significant anti-carcinogenic and immunity-stimulating effect [16]. Also, the *Pleurotus* species is rich source of some of the rare micro elements such as copper which has also reported on the surface of our synthesized nanoparticles and confirmed from XRD and EDS. The capping of pleuran on the surface of the nanoparticles was successfully achieved by using the developed method. This can be confirmed by our FTIR results which shows a clear prominent peak in the range (1620, 1440 and 1021 cm^−1^) which may be ascribed to presence of pleuran on the surface of nanoparticles.

During the synthesis process, the color changes indicated that the aqueous ME reduced the Ag^+^ into Ag^0^ and Au^3+^ into Au^0^. Mukherjee et al. [17] reported that reduction of metal ions in metal is due to the compounds; NADH reductases, Polychelatins and melanin present in fungal extract since the fungi *sajor-caju* is an aerobic fungus therefore it is obvious to have reductases in its extract. This enzyme was activated by microwave irritation and making them fast and efficient catalyst to reduce the precursor and indeed which was responsible for the process of oxidation and the reduction followed by the color changes, which is the indicator for Ag NPs and Au NPs synthesis. Further, on the scanning of NPs under UV-visible spectra showed maximum absorption peaks at 426 nm for Ag NPs and 531 nm for Au NPs (Figure 1b). The changes in color and appearance of the peak were due to the excitation of the SPR of Ag NPs and Au NPs. This particular analysis was confirmed and justified when a similar type of absorption spectrum was observed, which is obtained from the aqueous leaf extract of *Dendropanax morbifera* [18]. In another study, Mourato et al. [19] reported UV-Vis spectra of biosynthesized Ag NPs and Au NPs are 550 nm 420 nm, which is close to our finding. Approximate peaks i.e., 413 nm for Ag and 545 for Au were also reported by Philip et al. [20], He has also reported that levels of SPR peaks depend upon the concentration of ME added during the process. While stability of NPs depends upon the proteins bind on the surface of fungi which are present in bio-extract [21].

The XRD patterns (Figure 2) revealed the crystalline nature of biosynthesized Ag NPs and Au NPs with FCC structure, which is also supported by the TEM data and SAED pattern. The crystallite size from XRD data of Ag NPs and Au NPs is 16.82 nm and 16.90 nm, which was also supported by TEM images. One single small peak of 2θ = 30.64° also appeared due to the Cu present in the PS extract. This Cu interacts with nitrate ion of AgNO_3_ solution, forming Cu(NO_3_)_2_ whose peak was observed at 30.64° corresponds to its standard peak (114) (JCPDS No. 85-2019). It is clear from Table 2 that the calculated average crystallite size of Ag NPs and Au NPs is 16.82 nm and 16.90 nm, respectively, and was determined by the Scherrer’s equation from XRD data (Table 1). The phytochemicals, flavonoids and other secondary metabolites present in the fruiting body of oyster mushroom may have played a crucial role in the rapid biosynthesis of Ag NPs and Au NPs [22].

Recently, DLS has been considered as an alternative technique for the determination of the particle size of nanoparticles (Figure 3). Before carrying out DLS investigation, TEM was performed to analyse the size and size distribution of the Au NPs in nanofluid. From the DLS method, we determined the hydrodynamic diameter of biosynthesized nanoparticles which was greater than particle size obtained from TEM analysis [23,24]. The hydrodynamic diameter of nanofluid was measured 23 nm and 37 nm for Ag and Au nanofluid respectively. At the time of DLS measurement the size of the nanoparticles looked more significant than the TEM micrographs due to the solvent layers present around the nanoparticles [25].

To understand the interaction among different atoms and types of functional groups in synthesized NPs and ME, FTIR characterization was performed. The obtained FTIR results confirmed that the aqueous extract of *Pleurotus sajor-caju* has potential in performing the role of reducing agent and stabilizer of Ag NPs and Au NPs. The FTIR analysis of PS extract, Ag NPs and Au NPs exhibited the presence of characteristic peaks in all samples. The functional groups, bending and stretching peaks were observed, justified by the results of Jain et al. [26] and Farah et al. [27]. In all three FTIR spectra (Figure 4, Table 2), the –OH group peak is most prominent due to the presence of tannins, flavonoids and eugenol and the sharpening of –OH peak in NPs is the result of excess consumption of –OH group during the reduction process [28].

The SEM and HR-TEM images depicted the surface structure, size and morphology of biosynthesized NPs. The surface morphology of the respective Ag NPs and Au NPs capped *P. sajor-caju* were analysed using SEM, and the images are presented in Figure 5A. The images show that the NPs were spherical in shape [12]. The micrographs of the two biosynthesized NPs demonstrated their dispersal, spherical and smaller sized structure (Figure 5B,C). The average diameters of the silver and gold NPs were 15–20 nm and 16–18 nm, respectively. This is in agreement with the previous reports of Shweta et al. [5] and Prathna et al. [29]. Smaller size gold and silver NPs with average sizes of 14.6 and 11.35 nm were also synthesized by using leaf extract of Tulsi plant [26]. Such small particle size is very favorable for the anticancer activity as a reduced size increases the efficacy of NPs. In our TEM images, all the NPs showed uniform distributions due to the bioorganic capping agent of the PS extract. The active biomolecules and secondary metabolites present in PS extract stabilize the Ag NPs and Au NPs for a long duration and prevent them from aggregation [30]. The EDAX analysis (Figure 6) depicted the energy peaks for the silver and gold NPs and this peak was also analysed and justified by the previous report of Devi and Joshi [31].

The antiproliferative activity of PS extract and biosynthesized NPs was demonstrated against the HCT-116 cell line by morphological changes (Figure 7), MTT assay (Figure 8a), ROS generation (Figure 8b), and DNA fragmentation assay (Figure 8c). In our study we have reported the morphological changes in treated HCT-116 cell lines as compared to the control line. The treated cells lost their shape, cell adhesion capacity and shrunk, with visible DNA fragmentation. Treatment of HCT-116 cells at 100 µg/mL revealed that Ag NPs had the maximum cytotoxicity followed by PS extract and Au NPs. PS Similar changes have also been reported on different cancer cell lines after treatment with Ag NPs, which were synthesized using plant extracts [32]. Some previous findings also show the cytotoxic effects of biologically synthesized Ag NPs against cancer cell lines [32,33]. In previous few reports, it was showed that extracellular polysaccharides, polysaccharide, exopolysaccharide and mycelial polysaccharides of *sajor caju* origin have antiprolifartive and antitumor effects on HeLa cells, Hep-2 cells lines and sarcoma 180 of mice [34,35,36,37].

The effect of Ag NPs on the cellular ROS production was highest, while Au NPs and PS extract showed almost equal effects (Figure 8b). This intracellular ROS kills the cells by damaging mitochondrial membrane integrity and increasing oxidative DNA damage [38]. This finding was supported by previous reports [39,40,41,42,43]. Miethling-Graff et al. [44] have suggested that cellular uptake of NPs was size regulated [45]. A previous study illustrated that the smaller size, para-hydroxybenzoate tetrahydrate (SPHT) mediated synthesis of Ag NPs showed 50% inhibition of cell proliferation after 24 h of treatment in the human colon cancer cell lines, HCT-15 and HT-29 [46]. Satapathy et al. [47] found that plant-mediated biosynthesized Ag NPs had greater anti-proliferative activity upon HCT-116 cell line than the synthetic Ag NPs and AgNO3. It is well-known that the surface of bio-synthesized NPs is covered with biomolecules (proteins, carbohydrates, polyphenolic compounds, etc.) present in plant extract which makes NPs stable and biocompatible. Therefore, the above data, further confirms the capping of active ingredients on the surface of nanoparticles, due to which they have better activity than plant extract alone.

**Figure 9:**
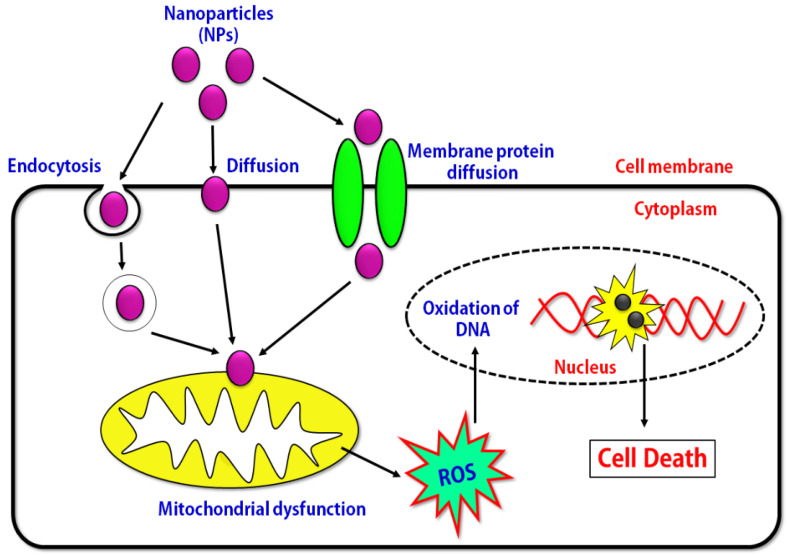
The proposed cytotoxic mechanism of *Pleurotus sajor-caju* medicated biosynthesized nanoparticles against colon cancer cells.

DNA fragmentation is a characteristic feature of apoptosis [48]. Two factors may be involved in the induction of apoptosis i.e., shrunken and irregular reduction in the size of cells, and DNA fragmentation (Figure 8c). In the present study, DNA fragmentation was confirmed by extracting DNA from HCT-116 cells treated with PS extract, Au NPs and Ag NPs followed by detection in the agarose gel, which is a signature of apoptosis (Figure 9). The deposition of NPs inside the nucleus could affect the DNA and cell division of the cells. Similar type of DNA laddering is observed in the evaluation of the toxic effect of Ag NPs against human breast cancer cell lines [48]. To summarize our developed method confirms the imprint of the active ingredient on the surface making it more competent to fight against the colon cancer cell line and thus opens the new era of herbonanoceutics.

## 4. Materials and Methods

### 4.1. Preparation of Spawn and Cultivation of the Mushroom, Pleurotus sajor-caju

The pure culture of *P. sajor-caju* was obtained from ICAR-DMR Solan, HP, India and was maintained in the Microbial and Mushroom Biotechnology laboratory of Centre of Biotechnology at the University of Allahabad (Allahabad, India). Spawn was prepared by inoculating vegetative mycelium of mushroom in glass bottles filled with half-boiled autoclaved wheat grains, which were buffered by mixing with CaCO_3_ and CaSO_4_ in 3:1 ratio. Thereafter, the inoculated spawn bottles were kept in a Bio-Oxygen Demand (BOD), incubator (METREX MRS-100c, New Delhi, India) at 25 ± 2 °C for 12–15 days to achieve full growth of milky white mycelia. To cultivate the mushroom fruiting body, the water-soaked sterilized paddy straw substrate was mixed with 5% spawn (1 kg wet weight of substrate mixed with 0.05 kg spawn) and packed in rubber tied polythene bags. After 20–25 days, fully grown fruiting bodies of mushroom were harvested and used for the synthesis of NPs and cytotoxic studies.

### 4.2. Preparation of Aqueous Extract of Pleurotus sajor-caju

The fully-grown fruiting bodies of *P. sajor-caju* were collected and decontaminated by washing with double distilled water. Then, 25 g of well washed fruiting bodies was crushed and homogenised in 75 mL of chilled triple distilled water with mortar pestle after that mixing of homogenate was done by stirring it on magnetic starrier (Remi-2MLH, Mumbai, India) for 36 h at 25 ± 2 °C. After mixing, homogenate was centrifuge (Remi-24BL) at 5000 rpm for 15 min at 4 °C and supernatant was collected and filter with Whatman filter paper No. 1 (pore size 125 mm). This aqueous filtrate used for the reduction of M^+^ ions to M^0^.

### 4.3. Extraction of Water-Soluble Substances from P. sajor-caju for Cytotoxic Study

For cytotoxic study of mushroom fruit body, aqueous cold extraction was done. In this extraction, 20 g of air-dried pieces of mushroom were powdered in Mixer Grinder (Philips HL1606/03 500 W, HP, India) and dissolved in 100 mL of distilled water followed by incubation in orbital shaker (Metrex, New Delhi, India) for 24 h at 25 ± 2 °C. This aqueous mixture of mushroom centrifuged at 5000 rpm for 15 min at 4 °C, supernatant was collected and filter by Whatman filter paper No. 1 (125 mm). The filtrate kept on rotatory evaporator (BUCHI, B-300, Mumbai, India) for the evaporation of water until the extract is appear like semisolid gel. This final extract was stored at 4 °C until further use.

### 4.4. Biosynthesis of Ag NPs and Au NPs

To synthesize Ag NPs and Au NPs, 100 mL of 0.01 M silver nitrate (AgNO_3_) and gold (III) chloride trihydrate (HAuCl_4_·3H_2_O) solution were prepared separately in triple distilled water and mixed well with a magnetic stirrer at 60 °C. During the synthesis of NPs, a quantity of reducing agent (PS extract) and temperature are important factor to controls the size of NPs. Therefore, the reducing agent was carefully added in drop by drop into the solution of AgNO_3_ and HAuCl_4_·3H_2_O and then was placed in a microwave oven (LG-MC3283AG, New Delhifor two min at 900 W under fast stirring conditions until the color changed to light brown and pinkish violet color, respectively. The color changes indicate the formation of Ag NPs and Au NPs. After synthesis, separation of NPs was achieved by stirring the colored mixtures overnight at room temperature, followed by centrifugation (12,000 rpm, 4 °C, 30 min). The supernatant was discarded and the pellets were washed several times with deionized water and dehydrated using pure ethanol and then used for further characterization and studies.

### 4.5. Characterization of Ag NPs and Au NPs

The optical properties of synthesized Ag NPs and Au NPs were confirmed by using a UV-Vis spectrophotometer (SPECORD 210 PLUS double beam spectrophotometer, Analytic Jena, Germany). Absorption spectra were measured in the range of 350–800 nm operated at a resolution of 1 nm to determine the corresponding λ_max_ values. The DLS was measured by the microtrack. Further, the ME and biosynthesized NPs were examined by FTIR spectra for the identification of functional groups in the transmittable mode ranging from 4000–500 cm^−1^ in KBr pellets (Thermo Scientific FTIR spectrometer, Waltham, MA, USA). The nature and purity of synthesized NPs were investigated by X-ray diffraction technique (XRD) (Proto A-XRD, Taylor, MI, USA) equipped with Cu Kα radiation λ = 1.5406 Å). The XRD study was done by using the films of the colloidal biosynthesized reaction mixture of Ag NPs and Au NPs formed on a microscopic glass slide by drop coating to confirm the crystallinity of biosynthesized NPs. The device operated at 40 kV, 40 mA, for 6 °/min, scanning rate, and 2θ diffraction angles ranged from 20° to 80°. Furthermore, the crystalline size of Ag NPs is calculated from XRD data using Debye Scherrer’s equation:

D=kλ/βs cosθ.

where *D* corresponds to the particle size, *k* is the shape-dependent Scherrer’s constant, λ is the wavelength of radiation, *βs* is the FWHM of the peak, and *θ* is the Bragg diffraction angle. The shape, size, morphology, and dispersal of the NPs were analysed by SEM (JEOL, model JSM6490LV, Akishima, Japan) EM, EDAX, elemental mapping and SAED pattern. This process used a carbon-coated copper grid on which one drop of biosynthesized Ag NPs and Au NPs was placed, dried at 60 °C, and observed under TEM-EDX microscopy (Tabletop Microscope Model TM 3000, Hitachi, Schaumburg, IL, USA).

### 4.6. Evaluation of Cytotoxicity of a ME (PS extract), Biosynthesized Au NPs and Ag NPs on HCT-116 Cell Line

The human colon cancer cell line (HCT-116) was purchased from the National Centre for Cell Sciences (Pune, India). The cytotoxicity of the aqueous PS extract and biosynthesized Ag NPs and Au NPs was tested on the HCT-116 cell line for determining the cell viability via MTT assay, ROS production and DNA fragmentation assay.

#### 4.6.1. Cell Culture

Human colon cancer HCT-116 cell line was cultured in Dulbecco’s modified Eagle’s medium (DMEM), supplemented with 10% fetal bovine serum and 1% penicillin/streptomycin at 37 °C in a humidified atmosphere of 5% CO_2_.

#### 4.6.2. Cell Viability Assay

The cytotoxic activity of the PS extract and biosynthesized NPs was tested against the HCT-116 cell line by a MTT (3-(4,5-dimethylthiazol-2-yl)-2,5-diphenyltetrazolium bromide) colorimetric assay. HCT-116 cells at a density of 0.5 × 10^4^ cells/well were plated in a 96-well plate and incubated for 24 h at 37 °C in 5% CO_2_ atmosphere. After 24 h, the cells were treated with different concentrations (20, 40, 60, 80, 100 µg/mL) of PS extract, biosynthesized Au NPs and Ag NPs with control. The cells were placed into an incubator and incubated for 24 h at 37 °C in 5% CO_2_ atmosphere. The cytotoxic behaviour of PS extract and biosynthesized Ag NPs and Au NPs on HCT-116 colon cancer cells was determined by MTT assay as previously described [49]. Absorbance was measured at 540 nm using a microplate reader and calculated from the following equation. The required concentration of samples for 50% cell inhibition i.e., IC_50_ values were calculated graphically. Percentage (%) cell viability = (absorbance of treated cells at 540 nm/absorbance of control cells at 540 nm) × 100. Additionally, assessment of cell morphology was made using a phase-contrast microscope (1X73, Olympus, Japan) and photographs were captured at 10× magnification.

#### 4.6.3. Measurement of ROS Production

Estimation of ROS was done by adapting the method of Wang and Joseph by using 2’,7’-dichlorodihydrofluorescein-diacetate (DCFH-DA) [50]. The HCT-116 cells were treated with the various concentrations of PS extract, Ag NPs and Au NPs for 24 h along with control. Then, samples were washed twice with phosphate-buffered saline (PBS) before and after the incubation with DCFH-DA dye at 37 °C for 30 min in dark. The fluorescence intensity was monitored as the rate of oxidation of the dye in the cells at an excitation wavelength of 495 nm and an emission wavelength of 537 nm.

#### 4.6.4. DNA Fragmentation Assay

HCT-116 cells (10^6^ cells) were seeded in a 6-well culture plate and treated with 100 µg/mL of PS extract, Ag NPs and Au NPs for 24 h. The effective concentration (100 µg/mL) was based on MTT assay. After 24 h of treatment, cells were harvested and centrifuged at 10,000 rpm for ten min at 4 °C as described by [48]. Firstly, cells were lysed in lysis buffer for 30 min and then were incubated with 0.1 mg/mL proteinase K and 0.2 mg/mL RNase for 1 hr at 60 °C. After that, the fragmented DNA was extracted with a 1:1 ratio of phenol and chloroform and run in 1% agarose gel containing 2% ethidium bromide (EtBr). The lanes 1, 2, 3 and 4 were loaded with control, PS extract, Au NPs and Ag NPs of 100 μg/mL concentration. The fragmented DNA was viewed by exposing the gel to UV-transilluminator and photographed.

#### 4.6.5. Statistical Analysis

All experiments were performed in triplicate. The results are presented as mean ± standard deviation. Statistical analysis was performed using GraphPad Prism 5.0 software (San Diego, CA, USA). All data were analysed by one-way ANOVA using the post hoc Dunnett’s test. *p* < 0.05 was considered to be statistically significant.

## 5. Conclusions

We have demonstrated the facile biosynthesis of Ag NPs and Au NPs using *Pleurotus sajor-caju* extract under ambient conditions. The synthesized Ag and Au NPs were spherical and presented a FCC crystal structure. The synthesized NPs were monodisperse, with a particle size of 15–20 nm and 16–18 nm for Ag NPs and Au NPs, respectively. The cytotoxic activity of the PS extract, biosynthesized Ag NPs and Au NPs showed promising results against a human colon cancer cell line (HCT-116). Our result suggest that the new adopted synthesis method ensures the dual benefit from phytoingredients which further enhances the effectiveness observed in human colon cancer cell lines in a dose-dependent manner. Moreover, the developed Ag NPs showed enhanced cytotoxic activity in comparison with PS extract and Au NPs. These NPs reduced the proliferation of a cancer cell line by generating a large amount of intracellular ROS. The results of the present study suggest that herbonanoceutics-mediated synthesis opens a new era for cancer therapeutic application which combines the active ingredients of plant extracts and the benefits of nanosystems. The overall results demonstrated that the biologically synthesized AgNPs have more antiproliferative activity in comparison with PS extract and Au NPs which may be due to induction of apoptosis in the HCT-116 cancer cell line. Moreover, our result confirms that the new adopted synthesis method ensures the dual benefits from phyto ingredients which furthers enhances the effectiveness of advanced nanomedicine. Further research is still needed to determine the anticancer mechanism of the biosynthesized NPs to optimize their physicochemical characteristics in order to improve their selectivity.

## Figures and Tables

**Table 1:** Crystallite size of Ag NPs and Au NPs.

S. No.	2θ	FWHM	Miller Indices	Crystallite Sizes	2θ	FWHM	Miller Indices	Crystallite Sizes
1	37.32	0.71757	111	12.20	37.56	0.71757	111	12.22
2	43.31	0.3519	200	25.38	44.59°	0.3519	200	25.49
3	63.73	0.6950	220	14.05	64.05°	0.6950	220	14.08
4	76.86	0.6764	311	15.66	76.78°	0.6687	311	15.84

**Table 2:** Functional groups associated with significant vibration bands in the mid-IR spectrum of *P. sajor-caju*, biosynthesized Ag NPs and Au NPs.

S.N.	Wavenumber Range (cm^−1^)	Wavenumber (cm^−1^) *P. sajor-caju*	Wavenumber (cm^−1^) Biosynthesized PS-Ag NPs	Wavenumber (cm^−1^) Biosynthesized PS-Au NPs	Band Assignment and Functional Groups
1.	3029–3639	3379.3	3421.5	3418.3	O-H stretching i.e., alcohol, phenolic compounds and groups N-H stretching (asymmetric) of Amide-A
2.	1583–1709	1650.2	1645.5	1674	(C=C stretch) and stretch of amides C=O stretching of ester group
3.	1400–1000	1406.0	1420.1	1422.1	A combination of hindered rotation and O-H bending
4.	1400–1000	1042.7	1021.7	1024.2	C-N stretching
